# Model of metabolic and temperature fluctuations in the brain

**DOI:** 10.1186/1471-2202-12-S1-P164

**Published:** 2011-07-18

**Authors:** Jan Karbowski

**Affiliations:** 1 Institute of Biocybernetics and Biomedical Engineering, Polish Academy of Sciences, 02-109 Warsaw, Poland

## 

A general theory of metabolic, hemodynamic, and thermodynamic fluctuations in the cerebral cortex is presented based on mesoscopic activities of neurons and synapses. Spectral functions of fluctuations in metabolic activity, cerebral blood flow, and tissue temperature are determined analytically and discussed with respect to underlying fluctuations in neural activity. The dependence of these fluctuations on general neuroanatomical organization is also discussed. It is found that spatial temperature correlations have a long-range character due to heat diffusion, even in the absence of such correlations in neural activities. Based on these results, it is suggested that experimental imaging of brain temperature distribution may be useful in determining local activities of neurons. The paper is an extension of the previous approach [1], where metabolic power of cortical neurons was determined analytically.
